# Mutagenic properties of dimethylaniline isomers in mice as evaluated by comet, micronucleus and transgenic mutation assays

**DOI:** 10.1186/s41021-018-0106-3

**Published:** 2018-08-22

**Authors:** Arihiro Kohara, Mariko Matsumoto, Akihiko Hirose, Makoto Hayashi, Masamitsu Honma, Takayoshi Suzuki

**Affiliations:** 1 JCRB Cell Bank, National Institutes of Biomedical Innovation, Health and Nutrition, Osaka, Japan; 2 Division of Risk Assessment, National Institute of Health Sciences, Kawasaki, Japan; 3 Division of Genetics and Mutagenesis, National Institute of Health Sciences, Kawasaki, Japan; 4 Division of Molecular Target and Gene Therapy Products, National Institute of Health Sciences, 3-25-26 Tonomachi, Kawasaki-ku, Kawasaki, 210-9501 Japan

**Keywords:** Dimethylaniline, Comet assay, Transgenic mutation assay, Micronucleus assay, Mouse, Mutation spectrum

## Abstract

**Background:**

The carcinogenic potential of dimethylaniline (DMA) isomers in rodents and humans has been previously reported, and there is sufficient evidence for the carcinogenicity of 2,6-DMA in experimental animals. The target organ of carcinogenesis of 2,6-DMA is the nasal cavity. In the current study, six DMA isomers, 2,3-, 2,4-, 2,5-, 2,6-, 3,4- and 3,5-DMA, were evaluated for mutagenic properties.

**Results:**

Male ddY mice (3/group) were treated intragastrically (i.g.) with 200 mg/kg of one of the six DMAs, and a comet assay was performed on samples of bone marrow, kidney, liver and lung at 3 and 24 h after the treatment. Positive responses were observed in the kidney, liver and lungs of mice from all of the DMA treatment groups after 3 h and in the bone marrow of mice treated with either 3,4- or 3,5-DMA after 3 h; however, these effects were diminished at the 24 h time point. The micronucleus induction in the bone marrow was analysed in the same mouse at 24 h after the treatment. No induction of micronucleated polychromatic erythrocytes was observed after treatment with any of the DMAs.

Male transgenic Muta™ mice (five/group) were treated i.g. with 2,5-, 2,6- or 3,5-DMA at 100 mg/kg bw weekly for 4 weeks, and the *lacZ* and the *cII* mutation frequencies were examined in the nasal cavity, liver and bone marrow at 7 days after the last treatment. Statistically significant increases in the mutation frequencies of the *lacZ* and/or *cII* genes were observed in the nasal cavity of 2,5-DMA or 2,6-DMA treated mice. Sequence analysis showed increased incidences of AT to GC and GC to TA mutations in the nasal tissues.

**Conclusions:**

These findings suggest that the carcinogenic activities of DMAs are associated with mutagenic events.

**Electronic supplementary material:**

The online version of this article (10.1186/s41021-018-0106-3) contains supplementary material, which is available to authorized users.

## Background

**Fig. 1:**
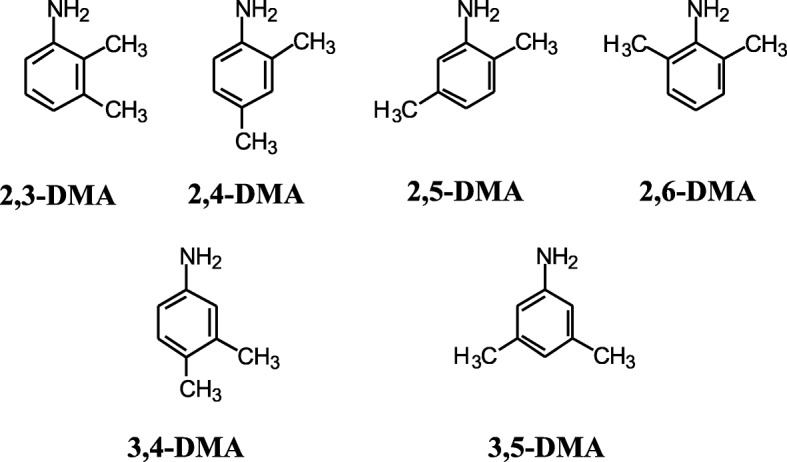
Structures of dimethylaniline (DMA) isomers

The structures of six dimethylaniline (DMA) isomers, a benzene-ring with two methyl functional groups and one amino functional group, are shown in Fig. 1. The carcinogenic potential of some DMAs has been previously reported. In a two-year carcinogenicity study, male and female rats that were fed diets containing 2,6-DMA at 3000 ppm showed significant increases in the incidences of adenocarcinomas or carcinomas of the nasal cavity and of the papillary adenomas of the nasal cavity, relative to control animals [1]. Rhabdomyosarcoma, a rare tumour of the nasal cavity, was observed in rats of each sex. Additionally, increased incidence of subcutaneous fibromas and fibrosarcomas in male and female rats and an increased incidence of neoplastic nodules of the livers of female rats were observed. In two-stage nasal carcinogenesis models using male rats, the tumour-promoting activity of 2,6-DMA was evident [2–4]. Another carcinogenicity study demonstrated that 2,4-DMA induced pulmonary tumours in female mice and that 2,5-DMA led to an increase in subcutaneous fibromas and fibrosarcoma in male rats and in vascular tumours in male mice [5].

Humans can be exposed to DMAs via various sources. For example, 2,6-DMA exists in tobacco leaves and is detected in tobacco smoke [6] and may also enter the environment through the degradation of some pesticides [1]. Some anaesthetics contain a DMA moiety; in particular, lidocaine is known to be metabolized to 2,6-DMA. After lidocaine treatment, 2,6-DMA-haemoglobin adducts and 2,6-DMA-DNA adducts in the urinary bladder were increased in humans [7, 8]. The use of permanent hair dye is associated with a level of 3,5-DMA-haemoglobin adducts [9]. Importantly, two epidemiologic studies conducted with six isomers of DMA or 2,6-DMA showed that levels of haemoglobin adducts of 2,6- and 3,5-DMA in peripheral blood samples were significantly associated with an increased risk of bladder cancer in humans [9, 10].

In bacterial reverse mutation assays with multiple strains of *Salmonella typhimurium* and *Escherichia coli*, positive or weak positive results were observed for 2,3-, 2,4, 2,5-, 3,4- and 3,5-DMA only when S9 mix was added [11–14]. Contrasting results were found for 2,6-DMA with or without the addition of S9 mix [1, 13, 15]; however, the most reliable study, conducted by OECD TG 471, showed a positive result with S9 mix [15]. Results of in vitro chromosomal aberration tests have been reported for 2,3-, 2,4-, 2,6-, 3,4- and 3,5-DMA [11, 12, 14–16], and clastogenicity was observed for 2,3-, 2,4-, 2,6- and 3,5-DMA with or without the addition of S9, but not in Chinese hamster lung or ovary cells treated with 3,4-DMA. Inductions of gene mutations by 2,6-DMA [17] or 2,6- and 3,5-DMA [18] were reported in the *gpt* gene of AS52 cells with human S9.

Although a wealth of in vitro data demonstrating the genotoxicity of DMAs exists, there are currently no reports of in vivo gene mutation assays for any of DMA isomers. A transgenic mouse model constructed to assay mutations in the target organs of carcinogenicity is useful to evaluate if chemical-induced carcinogenesis is related to mutagenic events. In the present study, the transgenic mouse mutation assay was conducted using Muta™ mice to assess in vivo mutagenicity of 2,5-, 2,6- or 3,5-DMA in the nasal cavity, liver and bone marrow. In addition, a comet assay was performed with the bone marrow, kidney, liver and lung of ddY mice at 3 and 24 h after treatment with the isomers 2,3-, 2,4, 2,5-, 2,6-, 3,4- and 3,5-DMA. The comet assay is useful to evaluate a broad spectrum of types of DNA damage. This paper reports the results of the transgenic mouse mutation assay and comet assay for DMAs. A bone marrow micronucleus assay in the ddY mice, and a peripheral blood micronucleus assay in the Muta™ mice were also conducted, the results of which are briefly reported in this paper.

## Methods

### Chemicals

2,3-DMA (CAS: 87–59-2), 2,4-DMA (CAS: 95–68 − 1), 2,5-DMA (CAS: 95–78-3), 2,6-DMA (CAS: 87–62-7), 3,4-DMA (CAS: 95–64-7) and 3,5-DMA (CAS: 108–69-0) were purchased from Wako Pure Chemical Industries, Ltd. (Osaka, Japan). The structures of these DMAs are shown in Fig. 1.

### Animals

The comet assay was performed using male ddY mice obtained from Japan SLC (Shizuoka, Japan). The bone marrow micronucleus assay was also conducted in these animals. The gene mutation assay was performed with male Muta™ mice, supplied by Covance Research Products (PA, USA). The peripheral blood micronucleus assay was also conducted in these animals. All animals were housed in a room maintained at 20–24 °C and 55–65% humidity with a 12 h light-dark cycle, fed commercial pellets (Oriental Yeast Industries Co., Tokyo, Japan) and given tap water ad libitum. Animal experiments were performed in accordance with the recommendations of the ethics committee of the institution. Dose (100, 200 mg/kg) were set based on the reported mouse LD_50_ of DMAs (250-1070 mg/kg) [19].

### Comet assay in ddY mice

Three ddY mice/group were treated with 0 (vehicle: olive oil) or 200 mg/kg of one of the six isomers of DMA by oral gavage at a volume of 10 ml/kg body weight. The animals were sacrificed by cervical dislocation at 3 h or 24 h after treatment, and the bone marrow, liver, kidney and lung were collected and immediately processed for the comet assay. The tissues were washed in saline, minced and suspended at a concentration of 1 g/ml in ice cold homogenizing buffer (HBSS with 20 mM EDTA, 10% DMSO, pH 7.5) and gently homogenized. The femoral bone marrow samples collected at 24 h after treatment were divided into two portions. One portion was suspended in chilled homogenizing buffer and used for the comet assay. The other portion was used for the micronucleus assay. The cell suspensions were appropriately diluted in chilled homogenizing buffer and subjected to the comet assay.

Normal melting point agarose (NMA: 1%, 250 μl) was placed on slides and dried overnight. The cell suspensions (10 μl) and 75 μl of 0.5% low melting point agarose (LMA) (37 °C) were placed onto the 1% NMA. The slides were covered with a cover slip and placed on a chilled plate to allow complete solidification of agarose. Finally, 75 μl of 0.5% LMP agarose was quickly layered in the same manner after removing the cover slip. The slides were immersed in freshly made ice-cold lysis solution (2.5 M NaCl, 100 mM EDTA, 10 mM Tris (pH 10.0), 10% DMSO and 1% Triton X-100) in the dark at 4 °C for at least 60 min. The slides were then placed in a horizontal electrophoresis tank containing electrophoresis buffer (300 mM NaOH and 1 mM EDTA; pH 13.0 and higher) for 10 min, allowing salt equilibration and further DNA denaturation before electrophoresis at 1 V/cm, 300 mA for 20 min. The slides were washed three times (10 min each) with chilled neutralizing buffer (400 mM Tris, pH 7.5). Following the third wash, the slides were stained with 20 μg/ml EtBr and covered with a cover slip. To prevent drying, the slides were stored in a humidified container until microscopic examination. The same electrophoresis unit and power supply were used throughout the study.

The slides were examined using a fluorescent microscope (Olympus, Tokyo Japan). All slides were coded and examined blindly. A total of 500 randomly selected cells were examined per sample (animal). The comets were classified into five categories according to the report by Kobayashi et al. [20], [Type 1: without tail (no damage), Type 2: with a small tail, Type 3: with tail showing evident migration, Type 4: with definite tale showing a consistent amount of fragments, Type 5: Almost all of the DNA is present in the tail (severe genetic damage)] depending on the fraction of DNA migrated out into the tail.

Statistical analysis was made on the incidences of type I cells against corresponding control by the Student’s t-test.

### Micronucleus assay in the bone marrow of ddY mice

A portion of the cell suspension from the bone marrow of the above-described ddY mice was smeared onto a clean glass slide. After air-drying, the bone marrow smears were fixed and stained with Giemsa. For each animal, 1000 polychromatic erythrocytes (PCE) were examined for the presence of micronuclei. The ratio of PCE to normochromatic erythrocytes (NCE) was determined.

### Transgenic mouse mutation assay in Muta™ mice

Based on the number of *ortho* substitution of aniline, 2,5- [1], 2,6- [2], and 3,5-DMA (0) were selected and subjected to the transgenic mouse mutation assay. Groups of five Muta™ mice were administered 2,5-, 2,6- or 3,5-DMA by gavage once a week for 4 weeks at a volume of 10 ml/kg, at 100 mg/kg. Separate groups of the vehicle control (olive oil) were treated and maintained in the same manner. The liver, entire nasal cavity and bone marrow were collected immediately after sacrificing the animals 7 days after the last treatment. The tissue samples were quickly frozen in liquid nitrogen and then stored at − 80 °C until analysis. The genomic DNA was extracted from each tissue by the standard phenol/chloroform method as previously reported [21]. Briefly, homogenized tissues were incubated with RNase and proteinase K, and genomic DNA was extracted using a phenol/chloroform mixture and chloroform followed by ethanol precipitation and then dissolved in TE-4 buffer (10 mM Tris-HCl at pH 8.0 containing 4 mM EDTA).

The bacteriophage lambda vectors were recovered by in vitro packaging reactions. The DNA solution was gently mixed with the Transpack packaging extract (Stratagene) and incubated at 37 °C for 1.5 h twice. The positive selections for *lacZ* and *cII* mutants were performed as previously reported [21] with a slight modification. As for the *lacZ* model, the phage solution was absorbed to *E. coli* C *(lac*^*−*^*, galE*^*−*^) at room temperature for 20–30 min. For the titration, appropriately diluted *E. coli* solutions were mixed with LB top agar (containing 10 mM MgSO_4_) plated onto dishes containing bottom agar and the number of plaques formed were counted. The remaining phage- *E. coli* solution was mixed with LB top agar containing P-gal and plated as described. The plates were incubated overnight at 37 °C for selecting *lacZ* mutants. As for the *cII* selection, the phage solution was absorbed to *E. coli* G1225 (*hfl*^*−*^) at room temperature for 20 min. Appropriately diluted *E. coli* solutions were mixed with LB top agar (containing 10 mM MgSO_4_) and plated onto dishes containing bottom agar and incubated at 37 °C for 24 h for titration. For the selection of *cII* mutants, the remaining phage- *E. coli* solutions were plated similarly but incubated at 25 °C for 48 h. The mutant frequency was calculated by the number of plaques on the selection plates divided by the total plaques.

### DNA sequence analysis

Sequencing of *cII* mutants was conducted as follows. The entire lambda *cII* region was amplified directly from mutant plaques by *Taq* DNA polymerase with primers P1; 5′-AAAAAGGGCATCAAATTAAACC-3′ and P2; 5′-CCGAAGTTGAGTATTTTTGCTGT-3′, as previously reported [21]. PCR products for 446 bp, involving the entire (294 bp) *cII* gene, were purified with a MicroSpin S-400 HR column (Amersham) and then used for a sequencing reaction with the Ampli *Taq* cycle sequencing kit (Perkin-Elmer Biosystem, Tokyo Japan) using the primer P1. The reaction product was purified by ethanol precipitation and analysed by the ABI PRISM™ 310 Genetic Analyzer (Perkin-Elmer Biosystems, Tokyo, Japan).

### Micronucleus assay in the peripheral blood of Muta™ mice

Blood samples were collected from the tail vein of the Muta™ mice used in the transgenic mouse mutation assay at 48 h after the first treatment and examined for micronucleated reticulocytes (MNRETs) using acridine orange supravital staining. In this experiment, 1000 reticulocytes (RETs) per animal were examined for the incidence of micronucleus.

### Statistical analysis

The significance of differences in the mutant frequency between the treated and control groups was analysed using Student’s t-test. Significance was indicated by *P* values < 0.05.

## Results

### Comet assay in ddY mice

**Fig. 2:**
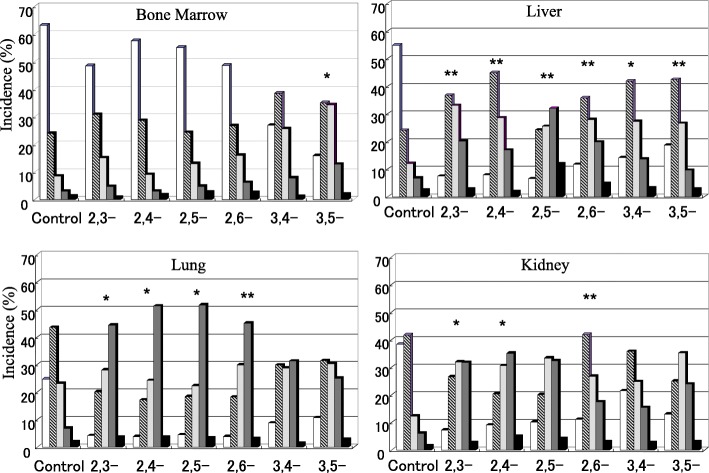
Comet induction in four organs (bone marrow, liver, lung and kidney) 3 h after the treatment of DMAs. Three ddY mice/group were treated with vehicle (olive oil) or 200 mg/kg of one of the DMA isomers by oral gavage. The animals were sacrificed at 3 h after treatment, and the bone marrow, liver, kidney, and lung were collected and processed for the comet assay. A total of 500 randomly selected cells were examined per sample. The comets were classified into five categories [ Type 1: without tail (no damage),  Type 2: with a small tail,  Type 3: with tail showing evident migration,  Type 4: with definite tale showing a consistent amount of fragments,  Type 5: Almost all of the DNA is present in the tail (severe genetic damage)] depending on the fraction of DNA migrated out into the tail. (* *p* < 0.05, **p < 0.01 for type I %)

**Fig. 3:**
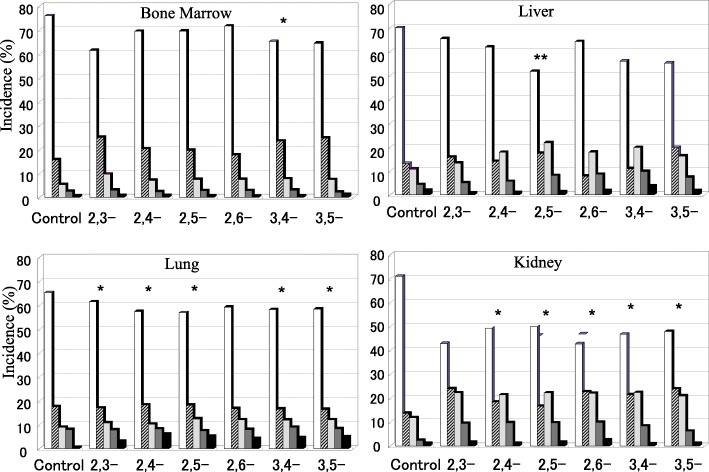
Comet induction in four organs (bone marrow, liver, lung and kidney) 24 h after the treatment of DMAs. Three ddY mice/group were treated with vehicle (olive oil) or 200 mg/kg of one of the DMA isomers by oral gavage. The animals were sacrificed at 24 h after treatment, and the bone marrow, liver, kidney, and lung were collected and processed for the comet assay. A total of 500 randomly selected cells were examined per sample. The comets were classified into five categories, [ Type 1: without tail (no damage),  Type 2: with a small tail,  Type 3: with tail showing evident migration,  Type 4: with definite tale showing a consistent amount of fragments,  Type 5: Almost all of the DNA is present in the tail (severe genetic damage)] depending on the fraction of DNA migrated out into the tail. (* *p* < 0.05, **p < 0.01 for type 1 %)

The distribution pattern of DNA damage in terms of DNA migration among the different treatment groups is expressed as the percent of cells in the five comet classes from Type 1 to Type 5, as presented in Figs. 2 and 3. At 3 h, the numbers of comet cells classified as Type 3 and/or Type 4 were increased in the kidney and lung for all of the DMAs. Type 2 and/or Type 3 cells were also increased in the liver for all DMA treatment groups and in the bone marrow for 3,4- and 3,5-DMA (Fig. 2). Among six DMA isomers, incidence of DNA damage was higher after treatment with 2,3-, 2,4- and 2,5-DMA in the kidney and 2,5-DMA in the liver. Incidence of DNA damage was lower in the lungs of mice treated with 3,4- and 3,5-DMA. At 24 h after treatment, the DNA damage was recovered in mice from all treatment groups (Fig. 3). Detailed data were available in Additional file 1.

### Micronucleus assay in the bone marrow of ddY mice

No changes in the frequency of micronucleated polychromatic erythrocytes were observed for all six isomers of DMA in the bone marrow of ddY mice (Table 1). The PCE/NCE ratios (mean ± SD) for the vehicle control, 2,3-, 2,4-, 2,5-, 2,6-, 3,4- and 3,5-DMAs were 1.1 ± 0.0, 0.8 ± 0.2, 1.1 ± 0.2, 1.1 ± 0.4, 1.0 ± 0.1, 1.1 ± 0.1 and 0.9 ± 0.2, respectively. A strong induction of met-haemoglobins was observed in mice treated with 3,5-DMA.

**Table 1:** Micronucleus induction in bone marrow of ddY mice 24 h after the treatment of 2,3-, 2,4-, 2,5-, 2,6-, 3,4- and 3,5-DMAs at 200 mg/kg bw

Substance	MNPCEs/1000 PCEs^a^			Mean ± SD	PCE/NCE
Control	1	2	1	1.3 ± 0.5	1.1 ± 0.0
2,3-DMA	0	1	1	0.7 ± 0.5	0.8 ± 0.2
2,4- DMA	0	0	2	0.7 ± 0.9	1.1 ± 0.2
2,5- DMA	0	0	0	0.0 ± 0.0	1.1 ± 0.4
2,6- DMA	1	2	1	1.6 ± 0.9	1.0 ± 0.1
3,4- DMA	0	2	1	1.3 ± 0.5	1.1 ± 0.1
3,5-DMA	0	0	2	0.7 ± 0.9	0.9 ± 0.2

*MNPCE* micronucleated polychromatic erythrocytes, *NCE* normochromatic erythrocytes

^a^data from three mice

### Transgenic mutation assay in Muta™ mice

The MFs in nasal tissues, liver and bone marrow induced by 2,5-, 2,6- and 3,5-DMA are shown in Tables 2, 3 and 4. The MFs of the *lacZ* genes in 2,5- and 2,6-DMA-treated mice, and the *cII* genes in 2,5-DMA-treated mice were significantly increased in the nasal tissues. The MF of the *cII* genes in the 2,6-DMA treatment group was also increased in the nasal tissues, but the change was not statistically significant. No changes were found on the MFs of the *lacZ* and *cII* genes in the liver for 2,5-, 2,6- and 3,5-DMAs. As for the bone marrow, an increase in the MF of the *lacZ* genes was observed in mice treated with 2,5-DMA, but these increases are not considered biologically significant because total plaques of four animals in the control group were inadequate for evaluation.

**Table 2:** Mutation frequency in the *lacZ* and *cII* genes from the nasal tissues of Muta™ mice treated with 2,5-, 2,6- and 3,5-DMAs

Animal ID		*lacZ*				*cII*			
		Total plaques^a^	Mutants	MF (× 10^6^)	Mean ± SD	Total plaques^a^	Mutants	MF (× 10^6^)	Mean ± SD
Control (olive oil)	41	(36000)	5	138.9		(59400)	4	67.3	
	42	(13000)	2	153.8		(10800)	1	92.6	
	43	373,000	19	50.9		399,600	10	25.0	
	44	306,000	9	29.4		321,000	5	15.6	
	45	477,500	14	29.3		396,600	7	17.7	
	**Total**	**1,205,500**	**49**	**40.6**	**36.6 ± 10.2**	**1,187,400**	**27**	**22.7**	**19.4 ± 4.1**
2,5-DMA	81	471,000	40	84.9		546,000	41	75.1	
	82	821,000	71	86.5		871,200	40	45.9	
	83	388,500	42	108.1		639,600	28	43.8	
	84	731,000	70	95.8		916,800	32	34.9	
	85	566,500	40	70.6		552,600	31	56.1	
	**Total**	**2,978,000**	**263**	**88.3**	**89.2 ± 12.4** ^**c**^	**3,526,200**	**172**	**48.8**	**51.2 ± 13.7** ^**b**^
2,6-DMA	91	172,500	9	52.2		261,000	6	23.0	
	92	260,000	26	100.0		429,600	13	30.3	
	93	391,000	26	66.5		493,200	46	93.3	
	94	(0)	0	0		(0)	0	0	
	95	252,000	29	115.1		379,200	21	55.4	
	**Total**	**1,075,500**	**90**	**83.7**	**83.4 ± 25.2** ^**b**^	**1,563,000**	**86**	**55.0**	**50.5 ± 27.5**
3,5-DMA	101	276,500	11	39.8		1,854,600	16	8.6	
	102	554,500	49	88.4		850,800	27	31.7	
	103	665,500	23	34.6		810,000	18	22.2	
	104	601,500	22	36.6		411,600	22	53.4	
	105	192,000	14	72.9		246,000	11	44.7	
	**Total**	**2,290,000**	**119**	**52.0**	**54.4 ± 22**	**4,173,000**	**94**	**22.5**	**32.1 ± 15.9**

^a^Data with at least 100,000 total plaques were evaluated by the statistical analysis and a parenthesis indicates exclusion

^b^Significantly different from the control (P < 0.05) by t-test

^c^Significantly different from the control (P < 0.01) by t-test

**Table 3:** Mutation frequency in the *lacZ* and *cII* genes from the liver of Muta™ mice treated with 2,5-, 2,6- and 3,5-DMAs

Animal ID		*lacZ*				*cII*			
		Total plaques^a^	Mutants	MF (× 10^6^)	Mean ± SD	Total plaques^a^	Mutants	MF (× 10^6^)	Mean ± SD
Control (olive oil)	41	418,500	44	105.1		1,114,800	44	39.5	
	42	233,000	21	90.1		1,129,500	33	29.2	
	43	709,500	66	93.0		1,346,400	45	33.4	
	44	444,500	63	141.7		2,203,200	173	78.5	
	45	1,057,500	104	98.3		2,798,400	77	27.5	
	**Total**	**2,863,000**	**298**	**104.0**	**105.7 ± 18.7**	**8,592,300**	**372**	**43.3**	**41.6 ± 18.9**
2,5- DMA	81	331,250	38	114.7		1,257,000	56	44.6	
	82	165,000	15	90.9		846,000	27	31.9	
	83	639,000	26	40.7		892,800	21	23.5	
	84	2,109,000	220	104.3		2,120,400	129	60.8	
	85	(87000)	10	114.9		129,000	6	46.5	
	**Total**	**3,348,750**	**310**	**92.6**	**87.7 ± 28.4**	**5,245,200**	**239**	**45.6**	**41.5 ± 12.8**
2,6- DMA	91	573,000	72	125.7		1,473,600	41	27.8	
	92	840,000	76	90.5		2,028,000	72	35.5	
	93	2,826,000	230	81.4		945,000	25	26.5	
	94	2,435,000	193	79.3		3,013,800	164	54.4	
	95	131,250	11	83.8		612,000	11	18.0	
	**Total**	**6,805,250**	**582**	**85.5**	**92.1 ± 17.2**	**8,072,400**	**313**	**38.8**	**32.4 ± 12.3**
3,5- DMA	101	1,053,000	100	95.0		2,167,200	72	33.2	
	102	277,500	24	86.5		1,095,000	30	27.4	
	103	2,111,000	170	80.5		2,426,400	95	39.2	
	104	2,723,000	234	85.9		2,522,400	129	51.1	
	105	1,641,000	184	112.1		1,960,800	71	36.2	
	**Total**	**7,805,500**	**712**	**91.2**	**92.0 ± 11.1**	**10,171,800**	**397**	**39.0**	**37.4 ± 7.9**

^a^Data with at least 100,000 total plaques were evaluated by the statistical analysis and a parenthesis indicates exclusion

**Table 4:** Mutation frequency in the *lacZ* and *cII* genes from the bone marrow of Muta™ mice treated with 2,5-, 2,6- and 3,5-DMAs

Animal ID		*lacZ*				*cII*			
		Total plaques^a^	Mutants	MF (×10^6^)	Mean ± SD	Total plaques^a^	Mutants	MF (×10^6^)	Mean ± SD
Control (olive oil)	41	123,000	4	32.5		125,400	1	8.0	
	42	(88500)	5	56.5		227,400	4	17.6	
	43	(33000)	0	0		326,100	12	36.8	
	44	(32500)	0	0		(37500)	1	26.7	
	45	(60500)	2	33.1		(87000)	2	23.0	
	**Total**	**337,500**	**11**	**32.6**	**32.5**	**803,400**	**20**	**24.9**	**20.8 ± 12.0**
2,5-DMA	81	(42000)	7	166.7		122,700	2	16.3	
	82	320,500	26	81.1		422,400	6	14.2	
	83	238,500	17	71.3		374,400	19	50.7	
	84	215,000	5	23.3		162,000	8	49.4	
	85	(71500)	9	125.9		105,300	3	28.5	
	**Total**	**887,500**	**64**	**72.1**	**58.6 ± 25.3**	**1,186,800**	**38**	**32.0**	**31.8 ± 15.7**
2,6-DMA	91	155,000	12	77.4		272,100	3	11.0	
	92	120,000	17	141.7		216,300	14	64.7	
	93	676,000	12	17.8		240,300	4	16.6	
	94	(95500)	1	10.5		(91200)	3	32.9	
	95	584,500	31	53.0		509,700	24	47.1	
	**Total**	**1,631,000**	**73**	**44.8**	**72.5 ± 45.2**	**1,329,600**	**48**	**36.1**	**34.9 ± 22.0**
3,5-DMA	101	793,500	13	16.4		392,700	13	33.1	
	102	250,000	12	48.0		263,400	7	26.6	
	103	(55000)	4	72.7		121,800	6	49.3	
	104	(70000)	2	28.6		(95700)	2	20.9	
	105	119,500	5	41.8		140,100	4	28.6	
	**Total**	**1,288,000**	**36**	**28.0**	**35.4 ± 13.7**	**1,013,700**	**32**	**31.6**	**34.4 ± 8.9**

MFs of *lacZ* gene were not statistically evaluated because total plaques of 4 animals in the control group were less than 100,000

^a^Data with at least 100,000 total plaques were evaluated by the statistical analysis and a parenthesis indicates exclusion

### DNA sequence analysis

Table 5 shows a summary of the *cII* mutation spectra induced in the nasal tissues by 2,5- and 2,6-DMA. The sequence analysis showed an increased incidence of AT to GC transitions and GC to TA transversions. At CpG sites, transition of C to T, observed in the control group, was reduced by 2,6-DMA treatment.

**Table 5:** Summary of *cII* mutation spectra induced by 2,5- and 2,6- DMAs in the nasal tissues

Mutation Class	Control	(%)	2,5-DMA	(%)	2,6-DMA	(%)
Base Substitution	36	(80)	50	(88)	34	(97)
Transitions	30	(67)	37	(65)	19	(54)
GC to AT	29 [20]^a^	(64)	27* [19]	(47)	14* [11**]	(40)
AT to GC	1	(2)	10*	(18)	5	(14)
Transversions	6	(13)	13	(23)	15	(43)
AT to TA	2	(4)	2	(4)	2	(6)
AT to CG	2	(4)	0	(0)	3	(9)
GC to TA	0	(0)	7*	(12)	6*	(17)
GC to CG	2	(4)	4	(7)	4	(11)
-1 frameshifts	2	(4)	4	(7)	0	(0)
+ 1 frameshifts	7	(16)	2	(4)	1	(3)
Deletion	0	(0)	0	(0)	0	(0)
Insertion	0	(0)	1	(2)	0	(0)
Complex	0	(0)	0	(0)	0	(0)
Total	45	(100)	57	(100)	35	(100)

^a^Numbers in blankets are at CpG sites

* *p* < 0.05, **p < 0.01 Significantly different from control frequency by Fisher’s exact test (two tailed)

### Micronucleus assays in the peripheral blood of Muta™ mice

No changes in the frequency of MNRET were observed for 2,5-, 2,6- and 3,5-DMA in the peripheral blood of Muta™ mice. The mean numbers of MNRET per 1000 RETs (mean ± SD) for the vehicle control, 2,5-, 2,6- and 3,5-DMA were 1.6 ± 0.9, 1.0 ± 0.7, 1.6 ± 0.9 and 3.0 ± 1.2, respectively.

## Discussion

In the comet assay conducted in ddY mice, DNA damage was observed in the lung, kidney and liver for all of the DMAs and in the bone marrow of mice treated with 3,4- and 3,5-DMA at the 3 h timepoint. However, these types of damage were recovered in all of the mice at 24 h. Przybojewska et al. (1999) reported that DNA damage was observed in the liver cells of B6C3F1 male mice exposed to 2,4-DMA (i.p. injection) at 100 and 200 mg/kg bw 16 h after administration [22]. In another comet assay reported by Sasaki et al. (1999), a gavage dose of 2,6-DMA resulted in a positive result in the migration of nuclear DNA from stomach and urinary bladder cells at 8 h, in brain cells at 3 and 8 h and in lung cells at 8 h and 24 h in ddY mice at 350 mg/kg bw [23]. These results indicate that DMA isomers cause DNA damage in various organs. In the current comet assay, all of the DNA damage was recovered within 24 h. This is inconsistent with a study conducted by Sasaki et al. (1999), in which a positive result was observed at 24 h in the lungs, similar to that described above [23]. The dosage difference (200 mg/kg vs 350 mg/kg) might affect the difference in recovery. DNA damage caused by DMAs in the lung may persist under certain conditions. It should also be noted that 2,4-DMA has been shown to cause pulmonary tumours in female mice [5].

In the current in vivo micronucleus assays, no changes in the frequency of micronuclei were observed for all tested isomers of DMAs in the bone marrow or peripheral blood in mice. In two previous bone marrow micronucleus assays [24, 25], treatment with 2,6-DMA did not increase the frequency of micronuclei in the bone marrow of mice and did not change the PCE/NCE ratio in ICR mice, which is consistent with our results. Although in vitro chromosomal aberration tests indicated clastogenicity of 2,3-, 2,4-, 2,6- and 3,5-DMA with or without the addition of S9 [11, 14–16], it can be concluded that DMAs are not clastogenic in haematopoietic systems in vivo at 200 mg/kg (20–80% of LD_50_)*.* The difference between in vitro and in vivo results might be explained by the concentration of active metabolites in the bone marrow because the PCE/NCE ratio was not changed.

In bacterial reverse mutation assays, positive or weak positive findings were observed for six isomers of DMA when S9 mix was added [11–13, 15]. DMAs undergo metabolic *N*-hydroxylation, and all six *N*-hydroxy-DMAs were mutagenic in *S. typhimurium* TA100 [26]. After *N*-hydroxylation, DMAs are further metabolized, and formation of haemoglobin adducts and DNA adducts has been observed in several previously conducted studies [7, 26–28]. The DNA adduct in the nasal cavity, the major target organ of carcinogenicity, has been shown to be higher than in other organs such as the liver, urinary bladder and testes after administration of 2,6-DMA [8, 29, 30]. Tydén et al. (2004) showed that the capacity of CYP enzymes to activate DMAs in the nasal mucosa was higher than other tissues, such as the liver and forestomach [31]. In the current transgenic mutation assay using Muta™ mice, the MFs of the *lacZ* and/or *cII* genes were significantly increased in the nasal cavity of mice treated with 2,5- and 2,6-DMA, but not in the liver. Our findings suggest that the carcinogenic activities in the nasal cavity are related to mutagenic events. This is the first report showing a positive response in the nasal tissue by the transgenic mouse mutation assay. It is important to use the target organs of carcinogenesis for analysis in this assay.

Sequence analysis showed an increased incidence of AT to GC transitions and GC to TA transversions in the nasal tissues of mice treated with 2,5- and 2,6-DMA. Multiplicity of 2,6-DMA or 3,5-DMA adducts have been found by in vitro investigations [27, 28]. The dA (2′-deoxyadenosine) adduct was the most abundant product of the reaction of *N*-acetoxy-2,6-DMA with DNA [28], while the dG (2′-deoxyguanosine) adduct was the major product of the reaction of *N*-acetoxy-3,5-DMA with DNA [27]. In the current study, transition of C to T, normally observed in the control group, was reduced at CpG sites by 2,6-DMA treatment, which suggested a biologically significant increase in the MF. It is possible that adenine adducts may induce AT to GC transitions and are thus involved in the carcinogenic activity observed as a result of 2,6-DMA treatment, although the structure of the DMA-adducts have not been identified. Because the AT to GC transition is relatively rare type of mutation, it can be used as a molecular signature after confirmation in other systems.

Although the MFs of the *lacZ* and/or *cII* genes were increased by 2,5- and 2,6-DMA treatment, no changes were observed in the nasal tissues as a result of 3,5-DMA treatment. This result may suggest that the *ortho*-substituted DMAs are more mutagenic than *meta*-substituted DMA in the nasal cavity of rats. Marques et al. (1997) also showed that the position of methyl substitutes in DMAs influenced the mutagenic potential in *S. typhimurium* TA 100 and the stability of adduct formations [26]. In the current comet assay, *meta*-substituted DMAs, 3,4- and 3,5-DMAs, and four *ortho*-substituted isomers showed differing activity of DNA damage. That is to say, only 3,4- and 3,5-DMA caused DNA damage in the bone marrow, while they were shown to cause relatively weak DNA damage in the lung. As for 2,4- and 2,6-DMA, a species difference in metabolic pathways was found between rats and dogs, and general toxicity for repeated dosing was stronger for 2,4-DMA in rats and for 2,6-DMA in dogs [32]. Because isomer differences and species differences likely play a role in DMA toxicity, further investigation is necessary to gain a better understanding of the relationship between DMAs and carcinogenicity in humans.

## Conclusions

DMA isomers caused DNA damage in various organs, as evaluated by the comet assay, and isomer-specific activity was found. In the in vivo gene mutation assay, 2,5- and 2,6-DMA increased gene mutations in the nasal cavity and increased the incidence of AT to GC transitions and GC to TA transversions, suggesting that carcinogenic activities of DMAs are associated with mutagenic events. The pre-existing data for genotoxicity of DMAs are conflicting and not clearly demonstrated the genotoxicity of DMAs. The current result of the transgenic mouse mutation assay emphasized an importance of the in vivo test at the target organ for carcinogenesis.

## Additional file

Additional file 1: **Table S1.** Types of comet induced by DMAs at 3 and 24 h after the treatment in bone marrow, liver, kidney and lung of ddY mouse. (XLS 123 kb)
